# Extracorporeal Cytokine Adsorption in Acute Cardiovascular Care: Pathophysiological Insights and Clinical Perspectives

**DOI:** 10.3390/biomedicines14020360

**Published:** 2026-02-04

**Authors:** Klevis Mihali, Lukas Harbaum, Birgit Markus, Georgios Chatzis, Nikolaos Patsalis, Styliani Syntila, Bernhard Schieffer, Julian Kreutz

**Affiliations:** Department of Cardiology, Angiology, and Intensive Care Medicine, Philipps-Universität Marburg, Baldingerstrasse, 35043 Marburg, Germany

**Keywords:** cardiogenic shock, post-cardiac arrest syndrome, temporary mechanical circulatory support, extracorporeal cytokine adsorption, hemoadsorption

## Abstract

**Background**: Cardiogenic shock (CS) and post-cardiac arrest syndrome (PCAS) are frequently associated with a systemic inflammatory response resulting from ischemia–reperfusion injury, endothelial dysfunction, and microcirculatory impairment. This inflammatory biology may be further amplified by temporary mechanical circulatory support (tMCS) through blood–surface interactions and shear-related hemolysis. Extracorporeal cytokine adsorption has therefore been proposed as an adjunctive strategy to attenuate hyperinflammation and facilitate shock reversal in selected patients. **Methods**: We conducted a narrative review, guided by a targeted PubMed and Scopus search and reference screening, to summarize the current pathophysiological concepts and clinical evidence on extracorporeal cytokine adsorption in CS-, PCAS-, and tMCS-supported states. **Results**: Across porous polymer hemoadsorption cartridges (e.g., CytoSorb^®^), membrane-based or hybrid filters with adsorptive properties (e.g., oXiris^®^), and selective approaches targeting inflammatory mediators (e.g., PentraSorb^®^ CRP), available studies most consistently report short-term physiological effects, including reduced vasopressor demand, improved metabolic stabilization, and modulation of inflammatory markers. However, evidence of benefits to clinically relevant endpoints remains inconsistent in various clinical settings, and randomized data are limited. **Conclusions**: Extracorporeal cytokine adsorption is a biologically plausible adjunct in inflammation-driven acute cardiovascular syndromes, but current evidence does not support routine use. Phenotype-guided patient selection, early timing, and adequately powered, mechanism-informed randomized trials are required to define clinical efficacy and safety in defined patient populations.

## 1. Introduction

Cardiogenic shock (CS), post-cardiac arrest syndrome (PCAS), and clinical scenarios requiring temporary mechanical circulatory support (tMCS) represent three closely related yet distinct entities that rank among the most challenging conditions in acute cardiovascular care. Although differing in their underlying pathophysiology, all three share common features of profound hemodynamic instability and frequently coexist in critically ill patients. Despite advances in intensive care, reperfusion strategies, and mechanical support technologies, mortality rates remain high. In CS related to acute myocardial infarction (AMI), 30-day mortality continues to exceed 40–50%, even with early revascularization, as evidenced by contemporary registries and population-based cohorts [1,2,3,4]. These persistently poor outcomes highlight that myocardial pump failure and maladaptive systemic inflammation with macro- and microcirculatory disturbances are both major factors in hemodynamic instability and multiple organ failure [5,6]. PCAS adds an additional layer of complexity: Global ischemia–reperfusion during cardiac arrest and resuscitation triggers a sepsis-like syndrome with profound immune activation, vasoplegia, microvascular dysfunction, and secondary organ injury, often compounded by myocardial stunning and hypoxic–ischemic brain injury [7,8]. Like in refractory CS, tMCS devices, including percutaneous microaxial flow pumps (mAFPs) and veno-arterial extracorporeal membrane oxygenation (VA-ECMO), can contribute to the hemodynamic stabilization and improvement of end-organ perfusion. However, device–blood interactions themselves, complement activation, shear-induced hemolysis, and ischemic complications may exacerbate cytokinaemia and endothelial damage [9,10]. Moreover, extracorporeal cardiopulmonary resuscitation (eCPR) using VA-ECMO is becoming more common in patients with refractory cardiac arrest to restore macrocirculation. However, even after blood flow is normalized, inflammation and downstream endothelial activation may persist. Against this background, interest has grown in adjunctive extracorporeal strategies aimed at attenuating hyperinflammation and its haemodynamic consequences.

**Figure 1:**
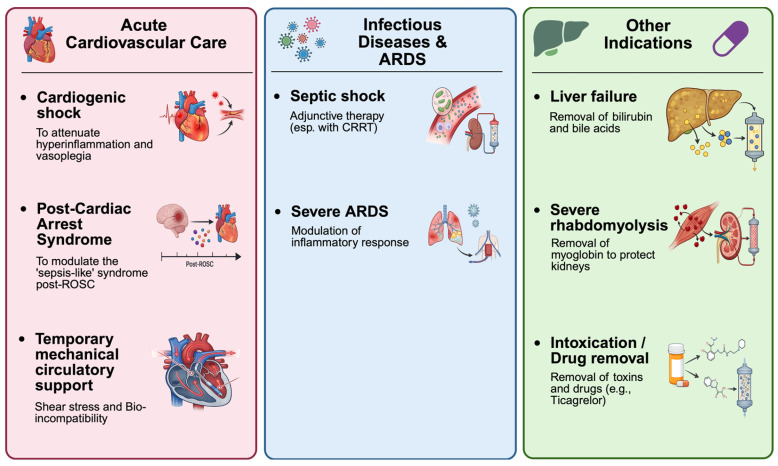
Overview of clinical contexts for extracorporeal cytokine adsorption in cardiovascular, septic, and toxin-associated critical illness. Created in BioRender. Kreutz, J. (2026) https://BioRender.com/6lkoaeu, accessed on 1 February 2026.

In acute cardiovascular intensive care, several extracorporeal cytokine adsorption approaches are currently applied to modulate systemic inflammation. These include classical cytokine adsorbers based on porous polymer resins, such as CytoSorb^®^ (CytoSorbents, Princeton, NJ, USA); membrane-based and hybrid filters with cytokine adsorption capacity, such as oXiris^®^ (AN69-oXiris, Baxter/Gambro [Vantive], Europe (Lessines, Belgium)/(Deerfield, IL, USA)); and selective adsorbers targeting other inflammatory mediators, such as PentraSorb^®^ CRP (Pentracor, Kleinmachnow, Germany). These devices can be used as stand-alone hemoperfusion systems or integrated into continuous renal replacement therapy (CRRT) and ECMO circuits. While extracorporeal cytokine adsorption has been extensively studied in sepsis and septic shock, its role in acute cardiovascular syndromes remains comparatively underrepresented and fragmented. In contrast to the large body of sepsis-focused literature, evidence on CS-, PCAS-, and tMCS-supported states is derived predominantly from small observational cohorts and selected randomized pilot trials. By aligning these syndromes within a shared immune–endothelial framework and highlighting device-specific inflammatory drivers, we aim to provide a cardiovascular-centred interpretive lens for patient selection, timing, and trial design. Across CS-, PCAS-, and tMCS-supported states, hemoadsorption has been associated with improved hemodynamic stabilization, reduced vasopressor demand, and modulation of inflammatory and metabolic markers in observational studies [11,12,13]. However, outcome benefits remain inconsistent and are not yet supported by randomized evidence. Accordingly, this narrative review integrates CS-, PCAS-, and tMCS-supported states into a shared immune–endothelial framework and contextualizes extracorporeal cytokine adsorption specifically within acute cardiovascular care. Rather than representing isolated indications, these conditions form a broader clinical continuum of hyperinflammatory critical illness in which extracorporeal cytokine adsorption has been explored across cardiovascular, septic, and toxin-driven contexts (Figure 1).

## 2. Methods

This article is a narrative review supported by identification of the targeted literature. To inform a concept-driven synthesis of pathophysiological mechanisms and clinical evidence, we consulted PubMed and Scopus (for publications from January 2018–December 2025) and complemented this by a reference screening of key articles and relevant reviews. The search terms were “cardiogenic shock” OR “post-cardiac arrest syndrome” OR “temporary mechanical circulatory support” OR “veno-arterial extracorporeal membrane oxygenation” OR “microaxial flow pump,” combined with “extracorporeal cytokine adsorption,” “hemoadsorption,” “CytoSorb,” “oXiris,” or “PentraSorb.” We focused on evidence from adult populations (≥18 years), reflecting the clinical scope of acute cardiovascular intensive care. Reference lists of key articles and relevant reviews were screened to identify the additional pertinent literature. No formal risk-of-bias assessment or quantitative evidence grading was performed, as these approaches are not appropriate for the integrative, narrative scope of this review.

## 3. Pathophysiological Basis of Hyperinflammation in Acute Cardiovascular Care

### 3.1. Cardiogenic Shock

CS is a state of critical end-organ hypoperfusion caused by primary cardiac pump failure, sustained by a maladaptive inflammatory response that disrupts macro- and microcirculatory coupling [14]. In AMI-related CS, regional ischaemia–reperfusion triggers cardiomyocyte necrosis, the release of damage-associated molecular patterns (DAMPs), the activation of innate immune pathways and a surge of cytokines such as tumour necrosis factor (TNF)-α, interleukin (IL)-1β and IL-6 [15,16]. Downstream, endothelial activation with glycocalyx shedding, junctional instability, and immunothrombosis promotes microvascular obstruction and impaired oxygen extraction, thereby perpetuating vasoplegia and progressive organ dysfunction despite the restoration of epicardial flow [17]. Similar immune–endothelial mechanisms operate in non-ischaemic CS aetiologies, including acute decompensated heart failure, fulminant myocarditis, stress-induced cardiomyopathy, right ventricular failure, and mechanical complications of AMI.

Guideline-directed management prioritizes the rapid identification and definitive treatment of the underlying cause, early revascularisation in AMI with culprit-lesion percutaneous coronary intervention (PCI) or urgent coronary artery bypass grafting (CABG) when indicated, and structured haemodynamic support [18]. In persistent shock, invasive monitoring can support the individualized titration of preload, afterload, and inotropy [19,20,21]. Norepinephrine is the first-line vasopressor to restore mean arterial pressure, while inotropic support with dobutamine or milrinone is used to augment cardiac output, tailored to blood pressure, arrhythmia risk, and renal function [22,23]. The use of tMCS is considered according to shock severity and trajectory, typically following the Society for Cardiovascular Angiography and Interventions (SCAI) shock stages, with escalation from percutaneous left ventricular unloading with mAFPs to VA-ECMO in refractory cases and combined strategies when needed for oxygenation and perfusion alongside ventricular unloading. Current guidelines recommend using mAFPs in selected patients with CS, and recent studies highlight the increasing evidence supporting its use in CS associated with AMI [24,25]. Concomitant therapies include guideline-based antithrombotic treatment, ventilatory optimization, prevention and treatment of arrhythmias, correction of electrolyte and acid-base disturbances, and timely surgery for mechanical complications [26,27]. Alongside these measures, a subset of patients remains vasoplegic with persistent microcirculatory dysfunction, providing the biological rationale for adjunctive inflammation-modulating approaches targeting immune–endothelial dysregulation.

### 3.2. Post-Cardiac Arrest Syndrome

PCAS is conceptualized as a composite entity comprising hypoxic–ischaemic brain injury, transient post-arrest myocardial dysfunction, a whole-body ischaemia–reperfusion response, and persistent precipitating pathology [28]. Unlike predominantly regional ischaemia in AMI-related CS, PCAS is triggered by circulatory arrest followed by reperfusion. Together, whole-body hypoxia, disruption of the gut barrier, and resuscitation-related tissue injury trigger the release of DAMPs and endotoxins, activate pattern-recognition receptors and the complement system, and provoke a sepsis-like inflammatory response [29]. Studies have shown a correlation between higher concentrations of IL-6, IL-8, and IL-10 in the early stages, as well as the terminal complement complex, and more severe vasoplegia, lower cardiac output, an increased need for circulatory support, and a higher 30-day mortality rate [30,31]. These findings highlight the significance of the inflammatory response as a prognostic factor. Furthermore, endothelial damage has been shown to be a central mechanism in the pathophysiology of PCAS. Experimental and clinical data further suggest that ischaemia–reperfusion drives immune–endothelial dysfunction and blood–brain barrier injury, thereby linking post-resuscitation vasoplegia to secondary neuronal damage [32,33]. Post-resuscitation care focuses on the rapid identification and treatment of the precipitating cause, restoration and titration of haemodynamics with vasopressors and inotropes, lung-protective ventilation with careful oxygen and carbon dioxide control, fever prevention as part of temperature management, early consideration of tMCS in refractory circulatory failure, and structured neuroprognostication [8]. Within this bundle, patients exhibiting refractory vasoplegia, capillary-leak physiology and biochemical hyperinflammation despite optimized care represent the phenotype in which adjunctive, mechanism-targeted immunomodulation, including hemoadsorption within protocols or trials, may be explored.

### 3.3. Temporary Mechanical Circulatory Support and eCPR

While tMCS devices stabilize macrocirculation, they also introduce inflammatory drivers specific to the device, including blood–surface interactions, complement activation, and shear-induced hemolysis [34]. In VA-ECMO, non-pulsatile flow and retrograde aortic perfusion alter endothelial mechanotransduction, whereas mAFPs may promote hemolysis due to shear and suction effects [35,36]. Shear stress contributes to the development of acquired von Willebrand factor abnormalities and hemolysis. This releases plasma-free hemoglobin and heme, which scavenge nitric oxide and impair endothelium-dependent vasodilation. With mAFPs, such as the left ventricular Impella (Abiomed, Danvers, MA, USA), the load on the left ventricular wall is reduced and subendocardial blood flow improves. However, shear and suction effects can also cause hemolysis, activate the complement system, and contribute to cytokine release [34]. Combined approaches using VA-ECMO and Impella (ECMELLA) aim to balance oxygen supply and systemic perfusion with active unloading. In the context of refractory cardiac arrest, eCPR with consecutive VA-ECMO cannulation restores global flow but is followed by a pronounced whole-body reperfusion response that overlaps with PCAS biology and may persist despite normalized macrocirculatory parameters [37]. Management in tMCS-supported CS and PCAS focuses on meticulous device optimization and complication prevention. In VA-ECMO, key priorities are adequate anticoagulation, the monitoring and mitigation of hemolysis, ventilatory strategies to limit pulmonary edema, and timely venting or unloading of the left ventricle when pulmonary congestion, distension or intracardiac stasis occur [38]. In mAFP-supported patients, correct positioning, suction avoidance, hemolysis surveillance, and stepwise flow titration are central. In combined ECMELLA configurations, coordinated flow targets seek to balance oxygen delivery with ventricular decompression while minimizing recirculation and limb ischaemia. In these settings, it is clinically important to recognize hyperinflammatory trajectories, particularly when vasoplegia and lactate non-clearance persist despite technically adequate support, as this provides the context in which adjunctive cytokine-modulating strategies may be considered within institutional algorithms or trials.

**Figure 2:**
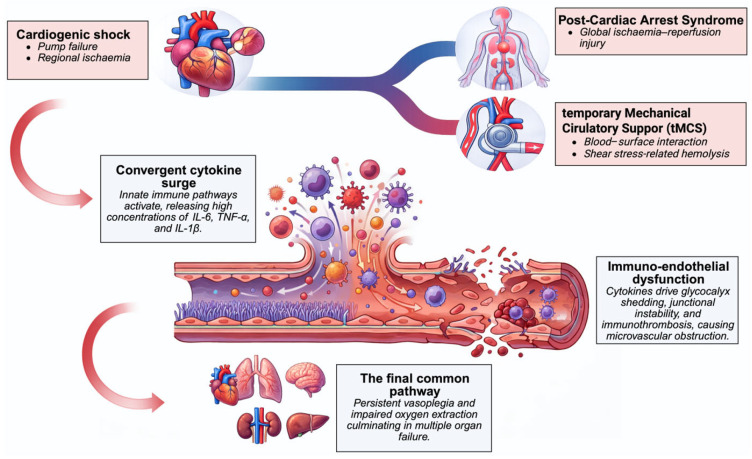
Convergent hyperinflammatory pathways in acute cardiovascular shock. Cardiogenic shock, post-cardiac arrest syndrome, and temporary mechanical circulatory support activate overlapping cytokine and endothelial injury cascades that culminate in vasoplegia, microvascular dysfunction, and multiorgan failure. Created in BioRender. Kreutz, J. (2026) https://BioRender.com/5bhc4hy, accessed on 1 February 2026.

Despite distinct initiating triggers, these syndromes converge on a shared hyperinflammatory end stage characterized by cytokinaemia, immune–endothelial dysfunction, microvascular failure, and progressive organ injury, forming a common biological substrate for adjunctive immunomodulatory strategies (Figure 2).

## 4. Rationale for Extracorporeal Cytokine Adsorption in CS, PCAS and tMCS

In the context of persistent immune–endothelial dysregulation despite macrocirculatory stabilization, extracorporeal cytokine adsorption has been proposed as an extracorporeal adjunct aimed at attenuating hyperinflammation rather than blocking individual signalling pathways [39]. The therapeutic goal is to mitigate endothelial activation and microvascular dysfunction, thereby improving vascular reactivity and macro–microcirculatory coupling by reducing the circulating concentrations of key mediators (e.g., IL-6, TNF-α, and IL-1β) as well as chemokines and alarmins [40]. In principle, this may translate into reduced vasoplegia, improved lactate clearance, and earlier de-escalation of vasoactive therapy; however, consistent effects on patient-centred outcomes have not been demonstrated [41]. Several blood purification approaches have been explored, including high-volume hemofiltration, high-cut-off membranes, plasmapheresis, and endotoxin-directed adsorption. Hemoadsorption is particularly attractive in cardiovascular intensive care medicine because it can be used as a stand-alone procedure or easily integrated into existing extracorporeal platforms, such as CRRT and VA-ECMO. It also covers a broad spectrum of cytokines and related mediators central to the biology of CS-, PCAS-, and tMCS-supported conditions [42].

## 5. Principles and Mechanisms of Extracorporeal Adsorption Therapies

The sorbent material, pore architecture, and surface functionalization of hemoadsorption systems differ, and together determine molecular breadth and adsorption kinetics [43]. In acute cardiovascular and intensive care medicine, the most used adsorbents are non-selective porous adsorption resins, such as CytoSorb. These cartridges typically contain cross-linked polystyrene–divinylbenzene beads with a hydrophilic coating and preferentially adsorb predominantly hydrophobic, mid-molecular-weight mediators (approximately 5–60 kDa) [44]. In addition to inflammatory mediators such as interleukins and TNF-α, resin cartridges can adsorb other clinically relevant solutes, including myoglobin and bilirubin, and may co-adsorb protein-bound medications [45]. This broad, concentration-dependent adsorption profile underlies their use in hyperinflammatory states and in device-supported shock, but it also necessitates careful consideration of drug exposure and dosing when adsorption is applied.

Membrane and hybrid designs expand the therapeutic spectrum for targeted applications. The oXiris^®^ membrane (Baxter, Deerfield, IL, USA) combines high-flow hemofiltration with coated adsorption surfaces, allowing for the simultaneous removal of cytokines and endotoxins [46].

PentraSorb^®^-CRP (PentraSorb GmbH, Kleinmachnow, Germany) is a highly selective adsorbent that targets the removal of C-reactive protein (CRP) to limit excessive complement activation and tissue damage [47].

Across platforms, clearance follows the rules of concentration and contact time. In acute cardiovascular care, integration into tMCS circuits and CRRT allows for early treatment. Outside of cardiovascular applications, hemoadsorption has been studied for use in septic shock, acute respiratory distress syndrome (ARDS) requiring venovenous extracorporeal membrane oxygenation (VV-ECMO), and in liver failure, rhabdomyolysis, and certain types of intoxication.

## 6. Clinical Evidence for Extracorporeal Cytokine Adsorption

### 6.1. Extracorporeal Cytokine Adsorption in CS- and tMCS-Supported Conditions

Beyond haemodynamic compromise, patients with CS frequently develop a systemic inflammatory response that contributes to vasoplegia, microcirculatory dysfunction, and secondary organ failure, providing the rationale for adjunctive extracorporeal immunomodulation.

In this setting, non-selective cytokine adsorption using CytoSorb has been most thoroughly studied. Across observational and propensity-matched cohorts of tMCS-supported CS, integration into VA-ECMO and/or CRRT has been associated with early improvements in haemodynamics and metabolic markers, including reductions in vasoactive–inotropic score (VIS), lactate, and inflammatory/organ injury markers such as CRP and procalcitonin (PCT) [11,48,49] (Table 1).

Although mortality remains high in refractory CS, in some cohorts, observed mortality was lower than predicted by risk scores; these descriptive comparisons are hypothesis-generating and cannot imply a causal treatment effect, and consistent survival benefit has not been demonstrated [45]. Feasibility and safety in complex extracorporeal settings have also been supported by broader single-centre experience and ECMO-focused reviews discussing implementation, controversies, and challenges [53,54,55]. Selected case reports further illustrate use in inflammatory cardiomyopathies and biventricular failure [56].

oXiris has been evaluated as an alternative adsorption platform in tMCS-supported CS, particularly in VA-ECMO populations. Evidence is limited to small, randomized studies showing transient reductions in inflammatory activity and vasoactive requirements (e.g., IL-6, VIS), without improvements in ECMO duration, weaning, organ dysfunction scores, or short-term survival [50,51]. Overall, current data do not support routine use of oXiris in tMCS-supported CS.

Selective C-reactive protein apheresis with PentraSorb has mainly been studied in cases of AMI cohorts. Although this method allows for quick and targeted removal of circulating CRP and shows biological effectiveness, clinical evidence is limited [52,57]. Its importance for the wider group of tMCS-supported CS patients seems minimal compared to non-selective cytokine adsorption strategies.

### 6.2. Extracorporeal Cytokine Adsorption in PCAS

Clinical evidence for cytokine adsorption in PCAS remains limited and inconsistent. Across available studies, non-selective adsorption with CytoSorb has been technically feasible and generally safe, both as stand-alone hemoperfusion and when integrated into VA-ECMO circuits during eCPR [13] (Table 2). In some cohorts, treatment was associated with reductions in inflammatory mediators, most notably IL-6, indicating biological activity [58]. Mechanistic data from eCPR populations further suggest that hemoadsorption may modulate leukocyte and platelet activation pathways, supporting target engagement beyond cytokine kinetics [59]. However, these signals have not translated into consistent improvements in haemodynamics, neurological recovery, or survival. Importantly, available observational data and randomized trials suggest that indiscriminate early use in unselected PCAS populations may be ineffective, and some datasets report an association with worse outcomes [60]. A plausible explanation is phenotype mismatch and timing: very early routine use in unselected patients may dilute any effect, whereas late initiation in advanced multiorgan failure may be biologically too late to modify outcomes. Registry-based analyses reported higher short-term mortality with early routine application, and randomized trials in eCPR patients did not demonstrate benefits in vasopressor requirements, markers of organ injury, or survival (Table 2). Taken together, current evidence supports feasibility and biological activity in selected patients but does not provide a consistent indication of clinical benefit on neurological outcomes or survival.

### 6.3. Extracorporeal Cytokine Adsorption in Septic Shock, Acute Respiratory Distress Syndrome, Rhabdomyolysis and Cardiac Surgery

Beyond acute cardiovascular syndromes, extracorporeal adsorption has been most extensively studied in sepsis and septic shock. In this context, CytoSorb has the most comprehensive clinical evidence and has been associated with reproducible short-term physiological effects, while a consistent survival benefit has not been demonstrated [61,62,63] (Table 3). Evidence for membrane-based adsorption systems such as oXiris is largely confined to septic shock with concomitant acute kidney injury during CRRT; small randomized and observational studies report reductions in inflammatory markers and vasopressor demand, but effects on mortality and duration of organ support remain inconsistent [64,65] (Table 3). Data supporting oXiris in ARDS are limited and have not shown robust clinical benefit beyond short-term physiological signals [66,67].

For rhabdomyolysis, registry data from the international prospective COSMOS registry suggest effective and safe reduction in very high myoglobin levels with CytoSorb, with concomitant improvement in creatinine and no serious device-related adverse events [78].

Selective CRP removal using PentraSorb has primarily been evaluated in cardiovascular indications; outside this context, extracardiac evidence remains scarce and has not demonstrated an impact on clinically relevant endpoints.

Other platforms (e.g., HA330/380 resin cartridges, endotoxin-directed adsorption, and pathogen-binding filters such as Seraph^®^ 100) show biological activity in selected sepsis-related cohorts, but robust outcome data are lacking.

In addition, hemoadsorption has been investigated in perioperative cardiac surgery during cardiopulmonary bypass (CPB), where contact activation can trigger a SIRS-like inflammatory response and vasoplegia. However, across a pilot RCT and subsequent observational data, intraoperative CytoSorb integrated into the CPB circuit has shown inconsistent effects on cytokine kinetics and has not translated into reproducible improvements in clinically relevant endpoints [79,81]. The largest randomized evidence in active infective endocarditis surgery (REMOVE trial) was neutral for postoperative organ dysfunction and 30-day mortality despite lower cytokine levels at the end of CPB [80].

Overall, extracardiac and perioperative data reinforce that adsorption therapies exert measurable biological effects, yet clinical relevance is indication- and phenotype-dependent and does not support routine use.

## 7. Practical Aspects in Extracorporeal Cytokine Adsorption: Patient Selection, Timing, and Dosing

**Figure 3:**
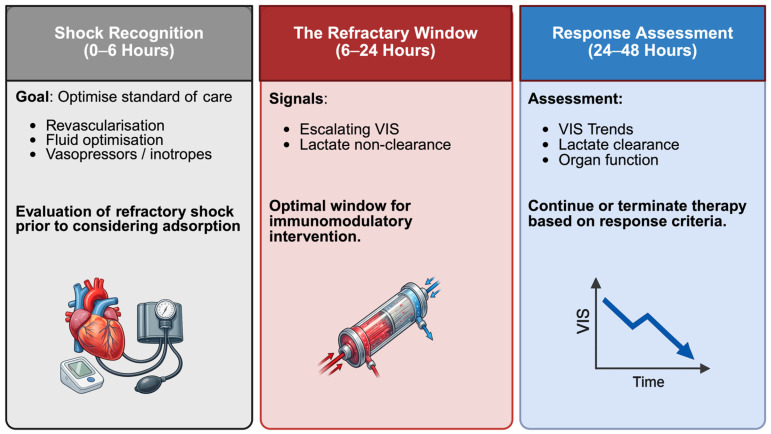
Phenotype-guided initiation and reassessment of extracorporeal cytokine adsorption in refractory cardiovascular shock, based on persistent hyperinflammation, metabolic non-clearance, and serial clinical response. Created in BioRender. Kreutz, J. (2026) https://BioRender.com/lpww9ll, accessed on 1 February 2026.

In acute cardiovascular care, hemoadsorption should be used as a time-critical, phenotype-guided adjunct within an optimized shock bundle. Candidate selection is trajectory-based: persistent vasoplegia with lactate non-clearance despite correction of the precipitating cause, technically adequate tMCS support, and optimized ventilation and volume strategy. A pragmatic treatable phenotype includes escalating vasoactive–inotropic requirements, metabolic non-clearance, and objective inflammatory activation. Initiation should be early after refractory shock persistence is recognized and competing causes have been excluded (e.g., bleeding, tamponade, inadequate unloading/flows, uncontrolled infection source, limb ischaemia). Treatment is typically provided in brief cycles involving standardized cartridge exchanges. Continued treatment depends on an early physiological response. Assessment of the response should be explicit and time-bound. This trajectory-based framework conceptualizes treatment as a sequence of defined therapeutic phases—initiation, early response assessment, and discontinuation—rather than a fixed-duration intervention (Figure 3). Meaningful response is suggested by a clear reduction in vasoactive support accompanied by improved lactate/acid-base status and stabilization of fluid balance and urine output within 24–48 h; in the absence of such a signal, early discontinuation should be favoured. Given the potential co-adsorption of hydrophobic/protein-bound drugs, protocols should address antimicrobial exposure and critical antiplatelet/anticoagulant/sedative dosing, including timing around cartridge changes and therapeutic drug monitoring when feasible [82]. The extent of clinically relevant drug removal is variable and remains incompletely characterized across settings, highlighting the need for therapeutic drug monitoring with feasible and standardized pharmacokinetic reporting in future trials. Safety monitoring should include circuit function and clotting, bleeding risk, hemolysis markers, and platelet trends. Since thrombocytopenia and hypoalbuminemia have been reported during extracorporeal therapies, platelet counts, and serum albumin levels should be monitored regularly [83].

## 8. Limitations

As a narrative review, this work is inherently selective and does not aim to be exhaustive. The current literature on extracorporeal hemoadsorption in acute cardiovascular intensive care is constrained primarily by study design and interpretability. Most data originate from retrospective, single-centre cohorts with small sample sizes. Propensity-based comparisons in these studies remain vulnerable to residual confounding factors and treatment selection effects, particularly in device-supported shock. Co-interventions in this setting vary substantially and may dominate outcomes. Even when short-term physiological signals are reported, randomized evidence in closely related settings such as eCPR/PCAS has not shown consistent benefit on patient-centred endpoints and has raised concern for harm when applied indiscriminately in unselected populations. Moreover, extracorporeal cytokine adsorption is not a single class intervention: CytoSorb, oXiris, and PentraSorb differ fundamentally in adsorption spectrum, kinetics, and circuit integration, limiting cross-device extrapolation and complicating synthesis across studies. Mechanistic studies support biological target engagement, but these signals remain only loosely linked to durable clinical outcomes.

Finally, the net clinical effect is difficult to quantify because non-selective adsorption may alter exposure to anti-infectives and other protein-bound drugs, and extracorporeal therapies may coincide with thrombocytopenia and hypoalbuminemia; standardized pharmacokinetic reporting, safety signal adjudication, and validated enrichment strategies (biomarker thresholds or composite phenotype definitions) are still lacking, limiting reproducibility and the precise identification of patients most likely to benefit. Future studies should predefine and report drug classes at risk (e.g., anti-infectives, antiplatelets, sedatives), timing relative to cartridge exchanges, and—where feasible—therapeutic drug monitoring to enable comparability and safety interpretation.

## 9. Conclusions and Future Perspectives

**Figure 4:**
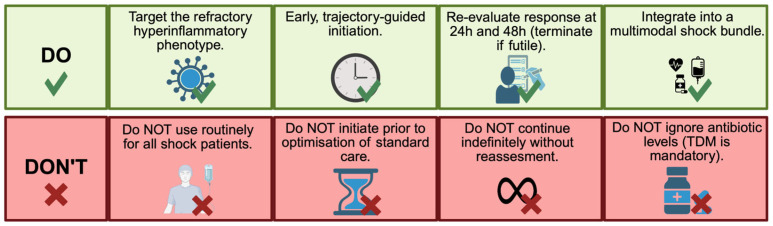
Practical do-and-do-not principles for phenotype-guided use of extracorporeal cytokine adsorption in acute cardiovascular care. Created in BioRender. Kreutz, J. (2026) https://BioRender.com/as8ywep, accessed on 1 February 2026.

Extracorporeal cytokine adsorption is a biologically plausible adjunct in acute cardiovascular shock syndromes in which immune–endothelial dysregulation contributes to vasoplegia and impaired tissue perfusion despite macrocirculatory stabilization. Across CS-, PCAS-, and tMCS-supported states, the available literature supports feasibility and short-term physiological effects in selected patients but does not justify routine use because consistent benefit on patient-centred outcomes has not been demonstrated. Accordingly, extracorporeal cytokine adsorption should be considered only as a selective, time-limited adjunct within multimodal shock care, guided by a clearly defined refractory, hyperinflammatory phenotype and continued only when an early, measurable physiological response is observed. In clinical practice, these principles translate into a structured set of indications and boundaries that emphasize selective use, early reassessment, and avoidance of indiscriminate application (Figure 4). Neutral or negative trial results may reflect phenotype mismatch, timing outside a therapeutic window (too early in unselected patients or too late in irreversible multiorgan failure), competing drivers of shock (e.g., inadequate unloading/flows, bleeding, ongoing ischemia), and variability in treatment “dose” and circuit integration. Future adequately powered, phenotype-enriched randomized trials are needed to define optimal timing and dosing and to evaluate clinically meaningful endpoints, including safety and drug-exposure effects.

## Figures and Tables

**Table 1:** Extracorporeal cytokine adsorption in cardiogenic shock supported with temporary mechanical circulatory support. HA: hemoadsorption; CS: cardiogenic shock; RCT: randomized controlled trial; VIS: vasoactive–inotropic score; ECMELLA: ECMO and Impella; VA-ECMO: veno-arterial extracorporeal membrane oxygenation; MCS: mechanical circulatory support.

Study Population	Methods	Hemoadsorption Protocol	Key Findings	Clinical Outcome
Soltesz et al., 2022–VA-ECMO + CytoSorb (n = 58) [48]	Propensity score matched cohort study (n = 29 each) in patients with refractory CS	VA-ECMO + CytoSorb, integrated into ECMO, replaced every 24 h, removed after 72 h	↓ SOFA score (*p* = 0.04), ↓ lactate (*p* = 0.015), ↓ *p*(v-a) CO^2^ gap (*p* < 0.001), ↓ CRP (*p* = 0.005), ↓ vasopressor requirement compared to control group	In-hospital mortality 44.8% vs. 62.1% (control), fewer bleeding complications in the HA group (*p* = 0.049); 90-day survival not significantly better
Lovrić et al., 2024–VA-ECMO + CytoSorb (n = 16) [49]	VA-ECMO-supported patients with CS, retrospective single-centre study	CytoSorb within the first 24 h after initiation of VA-ECMO	Significantly lower vasopressor doses, higher urine output, lower lactate levels and numerically lower mortality in the CytoSorb group (not statistically significant)	Mortality: 22.2% in the CytoSorb group vs. 57.1% in the control group (*p* = 0.12)
Kreutz et al., 2025–CS with CytoSorb (n = 129) [12]	Retrospective cohort study, CS patients, various types of MCS	CytoSorb in combination with MCS (Impella, VA-ECMO, ECMELLA)	Reduction in VIS from 38.0 to 16.3 (*p* = 0.002), reduction in lactate (*p* = 0.014), myoglobin (*p* < 0.01), LDH (*p* = 0.048) and PCT (*p* < 0.001); successful weaning from MCS	Observed in-hospital mortality (60.5%) was lower than predicted by the applied risk estimate (80%); hypothesis-generating. Improvement in organ perfusion and MCS support (not significant)
Ko et al., 2025–oXiris in patients with CS requiring VA-ECMO (n = 40) [50]	40 patients with CS and VA-ECMO, 20 with oXiris vs. 20 without	oXiris in combination with VA-ECMO	No significant difference in endotoxin levels at 48 h; significant temporal reductions in IL-6 (baseline-24 h *p* = 0.020; baseline-7 days *p* = 0.003); significant decrease in VIS at 48 h (*p* < 0.001) and 7 days (*p* = 0.007)	No significant differences in ECMO weaning, ECMO duration, or mortality
Nguyen et al., 2025–ECMORIX RCT (n = 40) [51]	40 patients with VA-ECMO and CRRT, 20 with oXiris, 20 with St-150 filter	oXiris in combination with VA-ECMO and CRRT	No significant differences in LPS plasma concentrations, no between-group differences in LPS activity, inflammatory markers (IL-6, TNF-α, IL-10, MCP-1), SOFA score, VIS	No difference in 28 days mortality
Torzewski et al., 2025–CRP-Apheresis in Acute Myocardial Infarction Registry [52]	Prospective registry-based case series; patients with NSTE-ACS treated with selective CRP apheresis in addition to standard-of-care therapy	Selective C-reactive protein apheresis initiated early after symptom onset; repeated apheresis sessions during the acute phase	Rapid and sustained reduction in circulating CRP levels; attenuation of inflammatory response; limited infarct size progression and preserved left ventricular function on follow-up imaging	Procedure feasible and safe with no treatment-related complications; survival of all treated patients during the acute phase

**Table 2:** Hemoadsorption in post-cardiac arrest syndrome. HA: hemoadsorption; CA: cardiac arrest; RCT: randomized controlled study; SOC: standard of care; eCPR: extracorporeal cardiopulmonary resuscitation; ECMO: extracorporeal membrane oxygenation; OHCA: out-of-hospital cardiac arrest; ROSC: return of spontaneous circulation; ICU: intensive care unit; NSE: neuron-specific enolase; S100b: S100 calcium-binding protein B (brain injury biomarker).

Study Population	Methods	Hemoadsorption Protocol	Key Findings	Clinical Outcome
Monard et al., 2021–PCAS (n = 21) [13]	RCT: Patients after CA, noradrenaline (>0.2 µg/kg/min) and/or serum lactate level > 6 mmol/L and/or time to ROSC > 25 min. 11 patients received HA, 10 patients received SOC	CytoSorb started within 18 h after admission to ICU, median duration 21 h	The median relative reduction in IL-6 after 48 h was 75% (60, 94) in the HA group compared to 5% (–47, 70) in the SOC group (*p* = 0.06)	HA was safe to administer to CA survivors at risk of PCAS
Supady et al., CYTER Trial, 2022–PCAS (n = 50) [58]	Single-centre, open label, randomized, controlled trial in patients scheduled for eCPR N = 26 with HA, n = 24 without	CytoSorb integrated into ECMO, changed every 24 h, removed after 72 h	IL-6 decreased in the CytoSorb group (408 → 324 pg/mL), while IL-6 increased in the control group (133→241 pg/mL); no significant difference	No significant improvement in survival, vasopressor requirement or biomarkers (NSE, S100b, troponin T)
Akin et al., HACORE Study, 2020 (n = 72) [60]	Patients after OHCA, HACORE database 24 patients with HA vs. 48 without HA	Early routine CytoSorb hemoadsorption (≤4 h after ICU admission)	No significant reduction in IL 6, but higher mortality in the hemoadsorption group (83% vs. 65%, *p* = 0.011)	HA appears to be associated with higher mortality after 30 days

**Table 3:** Hemoadsorption in extracardiac pathologies. HA: hemoadsorption; SOC: standard of care; ICU: intensive care unit; CRRT: continuous renal replacement therapy; ARDS: acute respiratory distress syndrome; vv-ECMO: venovenous extracorporeal membrane oxygenation; COVID-19: coronavirus disease 2019; PCT: procalcitonin; SOFA: Sequential Organ Failure Assessment; IL-6: interleukin-6, cardiopulmonary bypass (CPB).

Study Population	Methods	Hemoadsorption Protocol	Key Findings	Clinical Outcome
Hawchar et al., 2018: Extracorporeal cytokine adsorption in septic shock (n = 20) [68]	20 patients with early onset of septic shock without need for RRT, 10 with HA, 10 with SOC	Hemodialysis catheter inserted into central vein for CytoSorb	Significant decrease in norepinephrine (*p* = 0.016), PCT (*p* = 0.04) and Big-endothelin-1 (*p* = 0.03)	HA found to be safe with significant effects on vasopressor requirement, no effect on mortality (50% in both groups)
Brouwer et al.; 2019: Hemoadsorption with CytoSorb in ICU patients with septic shock (n = 116) [69]	Retrospective study, n = 67 patients with CRRT + CytoSorb, n = 49 with CRRT only	CytoSorb added to CRRT	Factors significantly associated with mortality at 28 days were SOFA score (*p* = 0.014), lactate levels (*p* = 0.014) and norepinephrine levels (*p* = 0.021). Days until start of CytoSorb in patients who died (1.13) vs. survival group (2.14)–not significant	Patients with CytoSorb had a significantly lower 28-day mortality (*p* = 0.038)
Schittek et al., 2020: Septic Shock and Acute Kidney Injury (SA-AKI) (n = 76) [70]	Retrospective control group and prospective intervention group in a tertiary hospital	Hemoadsorption used for patients with septic shock and SA-AKI	Patients treated with hemoadsorption had a shorter length of stay and reduced therapeutic support (e.g., catecholamine dependency, RRT duration). However, there were no significant differences in ICU or hospital mortality rates after multivariate analysis	No reduction in ICU or hospital mortality
Kogelmann et al., 2021: Early Septic Shock (n = 502) [71]	Retrospective analysis of 502 patients with septic shock, 98 received adjunctive CytoSorb treatment and 304 received standard therapy	CytoSorb adjunctive therapy, with timing and dosing guided by a dynamic scoring system	The dynamic scoring system identified patients with distinct mortality patterns. Early initiation of CytoSorb therapy significantly improved survival at 56 days, ICU, and hospital mortality	Early start of CytoSorb therapy was associated with significantly improved survival outcomes
Guan et al., 2022: CRRT with oXiris in AKI with septic shock (n = 136) [72]	Retrospective analysis of 136 patients with septic shock (n = 70) with oXiris, control group (n = 66) with the ST150 hemofilter	CRRT with endotoxic and cytokine adsorption function hemofilter (oXiris)	Early mortality in 7 and 14 days was significantly lower in oXiris group compared with ST150 group, significantly faster reduction in SOFA score, VIS and PCT after 24, 48 and 72 h	No difference was found in 90-day mortality, oXiris might reduce the short-term (<14-day) mortality compared with ST150 groups in septic shock with AKI
Broman et al., 2019: oXiris in septic shock (n = 16) [73]	Crossover double blind design, n = 9 patients with oXiris and CRRT, n = 7 with standard filter	CRRT in septic shock with oXiris membrane	Significant decrease in endotoxin levels, TNF-alpha, IL6/8 and IFNy, significant reduction in norepinephrine rate in oXiris patients	Mortality not described potential benefits in managing septic shock
Abdelaty et al., 2023: oXiris filter in critically ill COVID-19 patients (n = 58) [74]	National, multicenter, retrospective study of patients with COVID-19, Patients were categorized into two groups: Oxiris^®^ CRRT and standard CRRT	oXiris with CRRT in severely ill COVID-19 patients with AKI	Significant reduction in IL-6 and significant improvement in PaO_2_/FiO_2_ ratio after Oxiris^®^ CRRT initiation	Number of patients alive and ventilator-free at 30 days was higher in the Oxiris^®^ group, statistically not significant
Rieder et al., 2021: Severe ARDS requiring vv-ECMO (n = 18) [75]	Single-centre registry study comparing patients with and without cytokine adsorption, propensity score matching 9 Patients with HA and 9 without	CytoSorb hemoadsorption used in 9 patients with severe ARDS requiring vv-ECMO	Cytokine adsorption combined with vv-ECMO showed a numerical reduction in mortality and significant improvements in fluid resuscitation, vasopressor support, and lactate levels within 72 h	Mortality not described potential benefits in managing severe ARDS
Supady et al., 2021: CYCOV Study (n = 34) [76]	Single-centre RCT: patients with COVID-19 selected for ECMO, 17 with HA, 17 with SOC	CytoSorb device was incorporated into the ECMO circuit before connection to the patient circuit, replaced every 24 h, and removed after 72 h	No significant differences for IL-6 levels, lactate, fluid balance or survival	Early initiation of cytokine adsorption in patients with severe COVID-19 and vv-ECMO had a negative effect on survival
Akil et al.; 2022: Blood purification therapy in COVID-19 requiring vv-ECMO (n = 26) [77]	Retrospective study: 26 critically ill COVID-19 patients requiring vv-ECMO, 16 with HA, 10 with SOC	Hemoadsorption was either integrated into the ECMO circuit or into a CRRT machine run, adsorbers were changed every 24 h	Significant decrease in IL-6 in HA group, decrease in lactate (*p* = 0.067), norepinephrine levels were comparable after 72 h between the 2 groups	Mortality was similar in both groups, causes of death were not related to direct complication of vv-ECMO, CRRT or HA
Ferrer et al., 2025: Hemoadsorption therapy in severe rhabdomyolysis (n = 31) [78]	Observational, prospective, multicenter, international real-world data collection	Host circuit: RRT in 81%, stand-alone hemoperfusion in 19%. Median number of adsorbers per patient 3, mean daily adsorber usage 18.2 ± 9.9 h; mean total treatment duration 94.4 ± 133.3 h	Significant decrease in myoglobin (*p* = 0.001), creatine kinase (*p* = 0.002), lactate (*p* = 0.001), serum creatinine (*p* = 0.013), and 24 h fluid balance (*p* = 0.030)	ICU mortality 26%; no serious device-related adverse effects; platelets and albumin remained stable during therapy
Poli et al., 2019: Cytokine clearance with CytoSorb during cardiac surgery (n = 30) [79]	Single-centre pilot randomized controlled trial. Patients were randomly allocated to either SOC (n = 15) or CytoSorb (n = 15) during CPB	CytoSorb integrated into the CPB circuit (intraoperative only)	No significant decrease in measured pro-/anti-inflammatory cytokines across perioperative time points; no relevant adsorption of coagulation factors (only signs of coagulation activation)	Feasible/safe; no difference in vasopressor requirement, RRT, ICU length of stay, or in-hospital mortality
Diab et al., REMOVE trial, 2022: Cytokine hemoadsorption during cardiac surgery versus standard surgical care for infective endocarditis (n = 288) [80]	Multicenter randomized controlled trial, hemoadsorption vs. standard surgical care (138 vs. 142), four patients in the HA and 2 in the control group were excluded because they did not undergo surgery	CytoSorb integrated into CPB during surgery	Dampened surgery-associated cytokine increases lower selected cytokines at end of CPB, but primary endpoint (ΔSOFA) not different	Neutral, no reduction in postoperative organ dysfunction or clinically relevant secondary outcomes, including 30-day mortality

## Data Availability

No new data were created or analyzed in this study.
